# Neuron-secreted chemokine-like Orion interacts with the glial receptor Draper during mushroom body neuronal remodeling in *Drosophila*

**DOI:** 10.3389/fcell.2025.1664285

**Published:** 2026-01-20

**Authors:** Clarisse Perron, Ana Boulanger, Jean-Maurice Dura

**Affiliations:** IGH, Univ Montpellier, CNRS, Montpellier, France

**Keywords:** draper receptor, *Drosophila*, neuronal remodeling, neuron–glia crosstalk, Orion chemokine-like

## Abstract

Across the animal kingdom, neuronal remodeling is a crucial developmental mechanism to refine neurite targeting necessary for both maturation and function of neural circuits. The neuronal chemokine-like Orion is essential for astrocyte infiltration and likely for phagocytosis during mushroom body γ-neuron remodeling during metamorphosis in *Drosophila*. The Drpr phagocytic receptor is a critical and well-studied regulator of many aspects of neuronal remodeling, where it is required for neurite pruning and cell body removal. In this study, we show a *drpr* ^*null*^ allele displaying a mushroom body (MB)-pruning phenotype very similar, if not identical, to that of *orion* ^*null*^ alleles. Furthermore, when Orion is permanently tethered to the surface of the γ-axons, we show strong genetic interactions between neuronal Orion and glial Drpr. These results strongly suggest that Drpr is the glial receptor for Orion in mushroom body neuronal remodeling.

## Introduction

During nervous system development in both vertebrates and invertebrates, neurons undergo a remodeling process that is necessary for their normal function (Luo and O'Leary, 2005; Neukomm and Freeman, 2014; Schuldiner and Yaron, 2015). Moreover, it is suggested that excess or insufficient remodeling may underlie several neurodegenerative diseases (Cardozo et al., 2019; Courchesne et al., 2007). Glial cells play an essential role in both neuronal remodeling and neurodegenerative diseases. A critical crosstalk between glia and neurons is required in both processes; however, little is known about this intercellular cooperation (Boulanger and Dura, 2022; Neniskyte and Gross, 2017). Orion is a secreted chemokine-like protein, which has been described as a necessary component for neuronal remodeling in different paradigms in *Drosophila* (Boulanger et al., 2021; Ji et al., 2023; Perron et al., 2023). Orion is a ligand that should interact with its receptor to transform the surrounding glial or epidermal cells into phagocytes for neurite debris elimination. Therefore, the identification of the Orion receptor is of paramount importance to understand the phagocytic clearance of degenerating neurons. Importantly, in the case of the phagocytosis of degenerating dendrites of class IV dendritic arborization (C4da) neurons, it was shown that Orion bridges phosphatidylserine (PS), a conserved “eat-me signal,” and the phagocytic receptor Drpr (*draper)* (Ji et al., 2023). Drpr is involved in many aspects of neuronal remodeling and debris clearance during development or after injury in *Drosophila* (Boulanger and Dura, 2022; Freeman et al., 2003; Tasdemir-Yilmaz and Freeman, 2014). We aimed to assess whether Drpr can also be a receptor for Orion in the case of mushroom body (MB) neuronal remodeling. MBs are bilateral and symmetrical brain structures required for learning and memory. During metamorphosis, the MB-larval-specific dendrites and axons of early γ-neurons are pruned and replaced by adult-specific processes necessary for memory (Boulanger et al., 2011; Boulanger and Dura, 2015; Redt-Clouet et al., 2012; Trannoy et al., 2011; Yu and Schuldiner, 2014). We show here that the *drpr* ^*indel3*^ null allele has a MB-pruning phenotype very similar, if not identical, to that of *orion* ^*null*^ alleles. Furthermore, by using an Orion-CD2 transgene in which Orion is permanently tethered to the surface of the MB axons, we show, first, a high recruitment of glial Drpr inside γ-axon bundles and, second, strong genetic interactions between Orion and glial Drpr. These results strongly suggest that Drpr is the glial receptor for Orion in MB neuronal remodeling.

## Materials and methods

### *Drosophila* stocks

All crosses were performed using standard culture medium at 25 °C. All fly strains were tested, either through Western blotting, immunostaining, PCR or phenotypically. *orion* ^*null*^ mutants (*orion* ^*1*^ and *orion* ^*ΔC*^) were produced in a previous study in our laboratory (Boulanger et al., 2021). *drpr* ^*indel3*^, *UAS-orionB-CD2-mIFP*, and *UAS-HO1* flies were a gift from Chun Han. *drpr* ^*Δ5*^ and *UAS-drpr* flies were a gift from Marc Freeman. *Nimc1* ^*1*^ *; eater* ^*1*^ double-mutant flies were a gift from Bruno Lemaitre. *UAS>y* ^*+*^ *CD2>CD8-mGFP* (called here *UAS>y* ^*+*^ *>mGFP*) flies were a gift from Reinhard Stocker and used here, as *UAS-HO1*, as a neutral UAS to equilibrate the numbers of UAS sequences between experiments and controls. UAS-orionB-CD2-myc contains the same sequence, for the Orion-CD2 part, as UAS-orionB-CD2-mIFP previously described (Ji et al., 2023), and was synthesized by GenScript. Cloning and transgenesis were performed as in previous UAS constructions (Boulanger et al., 2021). We used two GAL4 lines: *201Y-GAL4* expressed in MB γ-neurons and *repo-GAL4* expressed in all glia.

### Brain dissection and immunostaining

Adult *drosophila* heads were fixated in formaldehyde (FA) 3.6% in phosphate-buffered saline (PBS) for 1 hour before dissection in PBS 1×. Larva were directly dissected in PBS 1×, and brains were then fixated in FA 3.6% for 15 min. They were then treated for immunostaining, as previously described (Boulanger et al., 2021). Antibodies were used at the following dilutions: mouse monoclonal anti-Fas2 (1D4, DSHB) 1:10; mouse monoclonal anti-Dally-like protein (Dlp) (13G8, DSHB) 1:200; rabbit monoclonal anti-myc (71D10, Abcam) 1:500; and mouse monoclonal anti-Drpr (8A1, DSHB) 1:300. Goat secondary antibodies conjugated to Cy3 and Cy5 against mouse and rabbit IgG, respectively (Jackson ImmunoResearch), were used at 1:300 for detection. For Western blotting, antibodies were used at the following concentrations: mouse monoclonal anti-Drpr (8A1, DSHB) 1:500; mouse monoclonal anti-tubulin (T5168, Sigma-Aldrich) 1:10,000; peroxidase-conjugated AffiniPure Goat anti-mouse IgG 1:10000 (Jackson ImmunoResearch).

### Western blotting

For protein extraction, 10 pupal brains (6 h APF) were mashed into 20 mL of Laemmli buffer 2× with β-mercaptoethanol for each condition and stored at −20 °C. Proteins were separated on a 10% agarose gel (Mini-PROTEAN TGX precast gels, Bio-Rad) and transferred to a PVDF membrane (Merck). The membrane was blocked in 5% milk before primary antibody addition overnight at 4 °C. After washing in PBS–Tween 0.1%, the membrane was incubated in milk 5% with the secondary antibody for an hour at room temperature and then washed again in PBS–Tween 0.1%. Detection was performed using ChemiDoc from Bio-Rad.

### Microscopy

Images were acquired using a Leica SP8 laser scanning confocal microscope (MRI Platform, Institute of Human Genetics, Montpellier, France) equipped with a 40× PLAN apochromatic 1.3 oil-immersion differential interference contrast objective lens. The immersion oil used was Immersol 518F. The acquisition software used was Leica Application Suite X (LAS X).

### Quantitation

For image quantitation of Orion-CD2 titration, MBs were analyzed using an epifluorescence microscope. They were blindly classified into two different groups based on the presence of the ''head'' of the MB dorsal γ-lobe (preserved) or absence of this head (headless). For Drpr titration, confocal images taken using the same confocal settings were used, and the surface of the dorsal γ-lobe was quantified using Fiji software. The dorsal lobe was first isolated; then, a threshold was manually set for each dorsal lobe, and the function ''Analyze particle'' generated the surface of fluorescence. To quantify the presence of Drpr in the mushroom body at larval stages, confocal images taken at the same confocal settings were analyzed on Imaris XT. A surface of the γ-lobe was created using the green fluorescence, allowing the generation of a second surface with the Drpr staining contained within it.

### Statistics

Comparison between two groups expressing a qualitative variable was analyzed for statistical significance using the Fisher exact test. Comparison of two groups expressing a quantitative variable was analyzed using the two-sided nonparametric Mann–Whitney U test. Graphs were created using GraphPad Prism software (version 10.3.1). Statistical significance was defined as ****P < 0.0001; ***P < 0.001; **P < 0.01; *P < 0.05; ns, not significant. The sample size of each group (n) is included in a parenthesis in figures.

### List of fly strains

Figure 1.

A. *y w* ^*67c23*^ */y w* ^*67c23*^ *; UAS-mCD8GFP 201Y-GAL4/+* or *y w* ^*67c23*^ */Y; UAS-mCD8GFP 201Y-GAL4/+*
B. *y w* ^*67c23*^ *sn* ^*3*^ *orion* ^*1*^ *FRT19A/Y; UAS-mCD8GFP 201Y-GAL4/+*
C. *y w* ^*67c23*^ */y w* ^*67c23*^ *; UAS-mCD8GFP 201Y-GAL4/+; drpr* ^*indel3*^ */drpr* ^*indel3*^ or *y w* ^*67c23*^ */Y; UAS-mCD8GFP 201Y-GAL4/+; drpr* ^*indel3*^ */drpr* ^*indel3*^
D–E. *y w* ^*67c23*^ */y w* ^*67c2*^ *; UAS-mCD8GFP 201Y-GAL4/+; drpr* ^*Δ5*^ */drpr* ^*Δ5*^ or *y w* ^*67c23*^ */Y; UAS-mCD8GFP 201Y-GAL4/+; drpr* ^*Δ5*^ */drpr* ^*Δ5*^
F. *w* ^*1118*^ *(Canton-S)*
G. *w* ^*67c23*^ *orion* ^*ΔC*^ */Y*
H. *y w* ^*67c23*^ */y w* ^*67c23*^ *; +; drpr* ^*indel3*^ */drpr* ^*indel3*^ or *y w* ^*67c23*^ */Y; +; drpr* ^*indel3*^ */drpr* ^*indel3*^
I–J. *y w* ^*67c23*^ */y w* ^*67c23*^ *; +; drpr* ^*Δ5*^ */drpr* ^*Δ5*^ or *y w* ^*67c23*^ */Y; +; drpr* ^*Δ5*^ */drpr* ^*Δ5*^
K. *w* ^*1118*^ *; Nimc1* ^*1*^ *; eater* ^*1*^
L. *y w* ^*67c23*^ */y w* ^*67c23*^ *or y w* ^*67c23*^ */Y*
*y w* ^*67c23*^ */y w* ^*67c23*^ *; +; drpr* ^*Δ5*^ */drpr* ^*Δ5*^ or *y w* ^*67c23*^ */Y; +; drpr* ^*Δ5*^ */drpr* ^*Δ5*^
*y w* ^*67c23*^ */y w* ^*67c23*^ *; +; drpr* ^*indel3*^ */drpr* ^*indel3*^ or *y w* ^*67c23*^ */Y; +; drpr* ^*indel3*^ */drpr* ^*indel3*^

**FIGURE 1:**
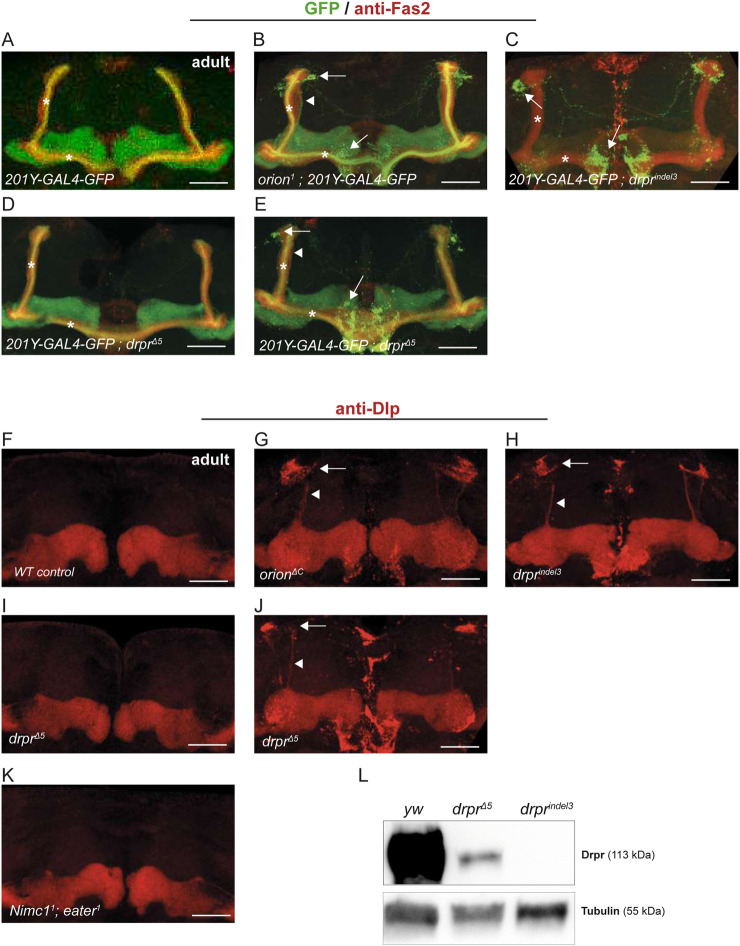
Drpr and Orion mutants exhibit a similar phenotype. **(A–E)** γ-neurons of 7-day adult flies were visualized via the expression of *201Y-GAL4*-driven *UAS-mCD8-GFP* (green) and using the anti-Fas2 antibody (red). For unknown reasons, the GFP fluorescence in the *201Y-GAL4-GFP; drpr* ^*indel3*^ strain appeared faint, except for the axonal debris that quickly reached saturation if increasing the signal. However, this effect is independent of the phenotype, which is clearly represented later in this figure with the anti-Dlp antibody. The *201Y-GAL4* line also labels the αβ-core axons shown here by asterisks. Note the presence of unpruned γ-axons (arrowhead) and the high amount of uncleared axonal debris (arrows) in *orion* ^*1*^, *drpr* ^*indel3*^, and *drpr* ^*Δ5*^ compared to the wild-type. *drpr* ^*indel3*^ MBs showed a lack of pruning with a complete penetrance (n = 36). All (100%) of these MBs showed both unpruned γ-axons and uncleared axonal debris as it is the case for the *orion* ^*1*^ MBs (n > 20). *drpr* ^*Δ5*^ MBs showed an intermediate phenotype: 85% of the MBs appeared wild-type, while 15% exhibited a mutant phenotype with at least some uncleared axonal debris (n > 20). A more detailed quantitation of unpruned axon and axon debris present in different genetic backgrounds can be found in our previous publication (Boulanger et al., 2021). **(F–K)** γ-neurons of 7-day adult flies were labeled with an anti-Dlp antibody (red). Unlike the previous labeling, this antibody labeled only the γ-neurons, making the unpruned axons and uncleared debris easily identifiable. With this labeling, 72% of *drpr* ^*Δ5*^ brains appeared wild-type, and 28% exhibited a mutant phenotype with at least unpruned axonal debris. *Nimc1* ^*1*^ *; eater* ^*1*^ brains were correctly pruned. n > 20 MBs for each condition. **(L)** Proteins were extracted from 10 whole pupae at 6 h APF for each condition, and Drpr expression was analyzed through immunoblotting. Note the complete absence of Drpr expression under the *drpr* ^*indel3*^ condition compared to *drpr* ^*Δ5*^. Scale bars represent 40 μm. All images are z-stacked confocal images. Immunostainings were replicated at least three times. Genotypes are listed in the complementary list of fly strains.

Figure 2.

- *y w* ^*67c23*^ *sn* ^*3*^ *orion* ^*1*^ *FRT19A/Y; UAS-mCD8GFP 201Y-GAL4/+; UAS-orionB-myc/+*
- *y w* ^*67c23*^ *sn* ^*3*^ *orion* ^*1*^ *FRT19A/Y; UAS-mCD8GFP 201Y-GAL4/+; UAS-orion-CD2-myc/+*

**FIGURE 2:**
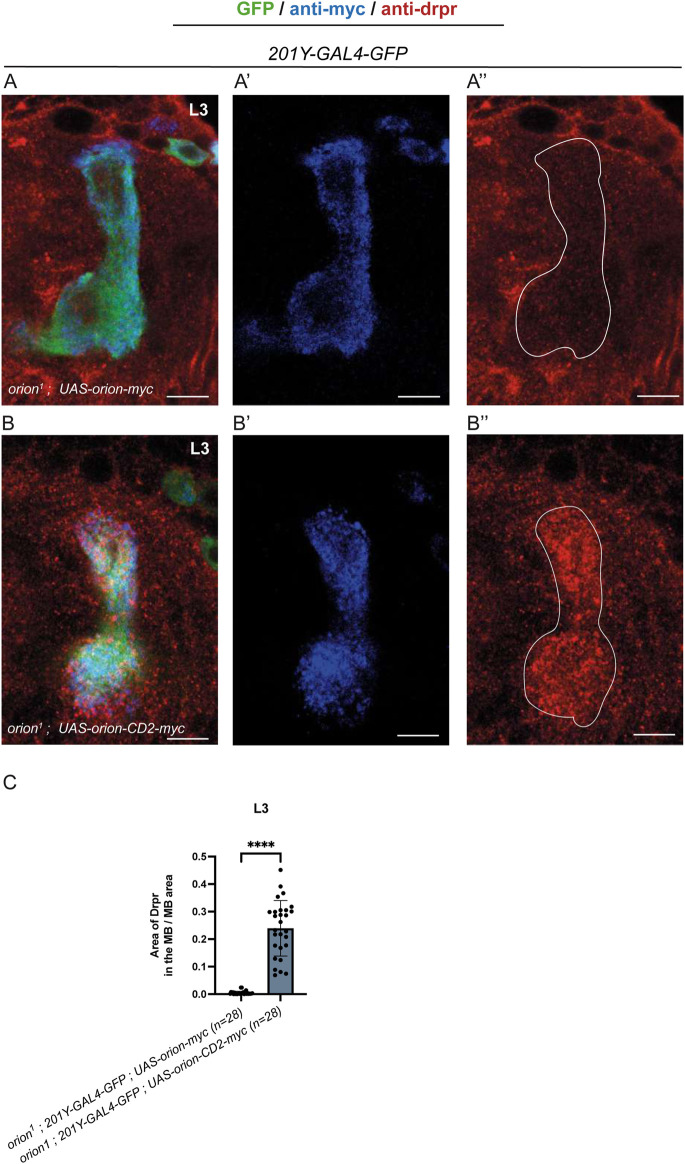
Orion tethered to the axon membrane recruits Drpr at the MBs. **(A,B)** Third-instar larval MBs were visualized using the expression of *201Y-GAL4*-driven *UAS-mCD8-GFP* (green). *UAS-Orion-myc* (A′) and *UAS-Orion-CD2-myc* (B′) were visualized with an anti-myc antibody (blue), and Drpr (A″, B″) was labeled with an anti-Drpr antibody (red). Note the complete absence of Drpr staining inside the MB in controls (A″) compared to the high amount of Drpr in the γ-lobe when Orion-CD2 is expressed in MBs and presented at the axonal membrane. **(C)** Quantitation of the Drpr area inside the MB γ-lobes. Values were normalized with the MB’s area and given as a ratio of “*Drpr area inside the MB/MB area.” n* values are indicated in the parenthesis and represent the number of MBs visualized. Error bars represent mean ± S.E.M. ****P < 0.0001 (Mann–Whitney U test). Immunostainings were replicated three times. Scale bars represent 10 μm. Images are single-slice confocal images, but the entire stack was analyzed. Genotypes are listed in the supplementary list of fly strains.

Figure 3.

- *y w* ^*67c23*^ */Y; UAS-mCD8GFP 201Y-GAL4/CyO* or *y w* ^*67c23*^ */Y; UAS-mCD8GFP 201Y-GAL4/UAS-mCD8GFP 201Y-GAL4*
- *y w* ^*67c23*^ */y w* ^*67c23*^ *; UAS-mCD8GFP 201Y-GAL4/+; UAS-orion-CD2, UAS > y* ^*+*^ *>mGFP/+* or *y w* ^*67c23*^ */Y; UAS-mCD8GFP 201Y-GAL4/+; UAS-orion-CD2, UAS > y* ^*+*^ *>mGFP/+*
- *y w* ^*67c23*^ */y w* ^*67c23*^ *; UAS-mCD8GFP 201Y-GAL4/UAS-drpr; UAS-orion-CD2/+* or *y w* ^*67c23*^ */Y; UAS-mCD8GFP 201Y-GAL4/UAS-drpr; UAS-orion-CD2/+*
- *y w* ^*67c23*^ */y w* ^*67c23*^ *; UAS-mCD8GFP 201Y-GAL4/UAS-drpr* or *y w* ^*67c23*^ */Y; UAS-mCD8GFP 201Y-GAL4/UAS-drpr*

**FIGURE 3:**
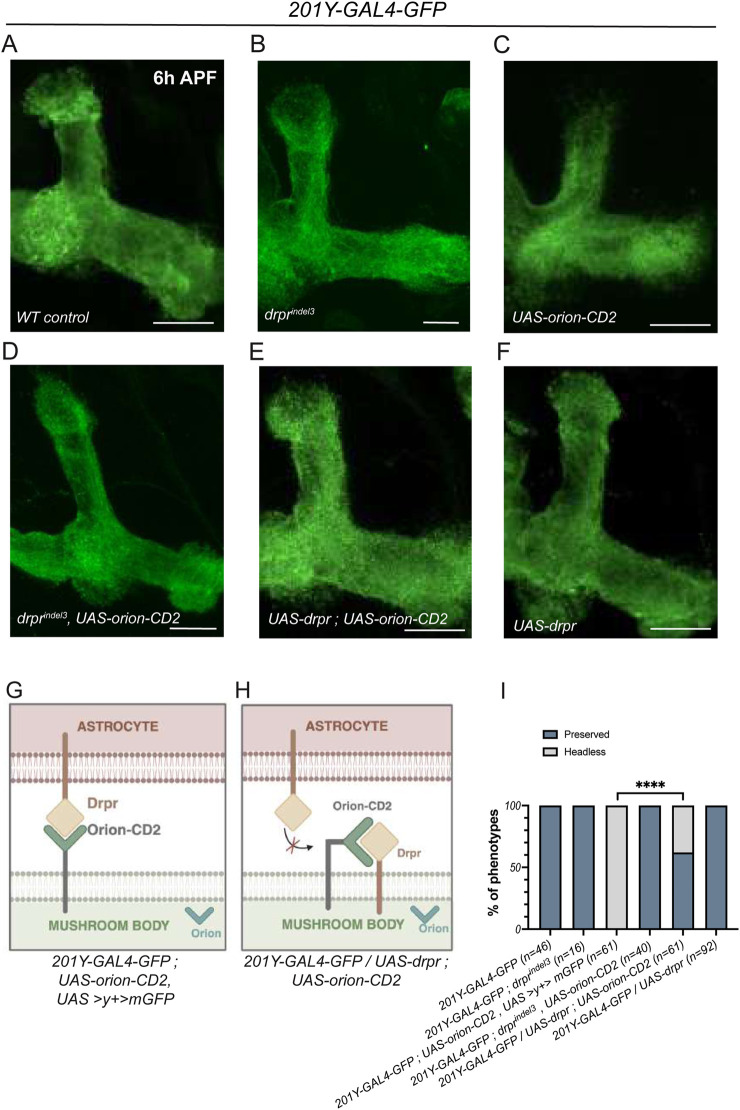
Drpr titrates membrane-tethered Orion in MBs. **(A–F)** γ-neurons of pupae at 6 h APF were visualized using the expression of *201Y-GAL4*-driven *UAS-mCD8-GFP* (green). Compared to controls **(A)** and to *drpr* ^*indel3*^ **(B)**, the expression of a membrane-tethered Orion-CD2 (along with a neutral *UAS > y* ^*+*^ *> mGFP*) in the MBs induces an earlier pruning and a characteristic ''headless'' phenotype **(C)**. This headless phenotype is rescued both by *drpr* ^*indel3*^ **(D)** and by overexpression of Drpr in the MBs **(E)**, suggesting an interaction between the two proteins. An overexpression of Drpr alone in the MBs does not induce any phenotype **(F)**. **(G,H)** Models of interactions between Drpr and Orion-CD2 (before endogenous Orion secretion). When Orion-CD2 is expressed in the MBs, it interacts with astrocytic Drpr and induces a ''headless'' phenotype at 6 h APF **(G)**. Overexpression of Drpr in the MBs leads to Orion-CD2 titration and prevents its interaction with endogenous astrocytic Drpr, resulting in the rescue of the pruning phenotype **(H)**. **(I)** Quantitation of the number of “preserved” and “headless” dorsal γ-lobes in MBs at 6 h APF for each condition. *n* values are indicated in the parenthesis and correspond to the number of MBs. Experiments were replicated three times. ****P < 0.0001 (Chi-squared test). Scale bars represent 20 μm. Images are z-stacked confocal images. Genotypes are listed in the complementary list of fly strains. Figures **(G,H)** were created with BioRender.com.

Figure 4.

- *y w* ^*67c23*^ *sn3 FRT19A/y w* ^*67c23*^ *; +; repo-GAL4 UAS-mCD8-GFP/+* or *y w* ^*67c23*^ *sn3 FRT19A/Y; +; repo-GAL4 UAS-mCD8-GFP/+*
- *y w* ^*67c23*^ */y w* ^*67c23*^ *; +; repo-GAL4/UAS-orion-CD2 UAS-HO1* or *y w* ^*67c23*^ */Y; +; repo-GAL4/UAS-orion-CD2 UAS-HO1*
- *y w* ^*67c23*^ */y w* ^*67c23*^ *; UAS-drpr/+; repo-GAL4/UAS-orion-CD2* or *y w* ^*67c23*^ */Y; UAS-drpr/+; repo-GAL4/UAS-orion-CD2*
- *w* ^*67c23*^ *orion* ^*ΔC*^ */Y*
- *y w* ^*67c23*^ */y w* ^*67c23*^ *; +; drpr* ^*indel3*^ */drpr* ^*indel3*^ or *y w* ^*67c23*^ */Y; +; drpr* ^*indel3*^ */drpr* ^*indel3*^
- *y w* ^*67c23*^ */y w* ^*67c23*^ *; UAS-drpr/+; repo-GAL4/+* or *y w* ^*67c23*^ */Y; UAS-drpr/+; repo-GAL4/+*

**FIGURE 4:**
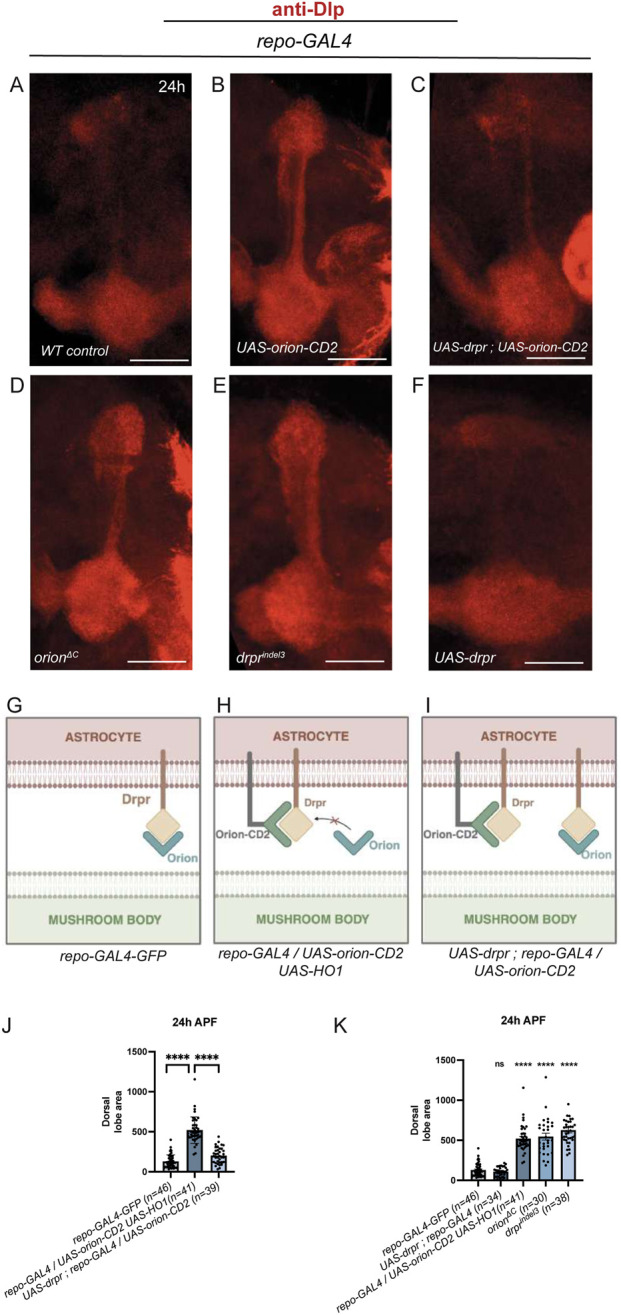
Membrane-tethered Orion titrates Drpr in astrocytes. **(A–F)** Dorsal γ-lobes of pupae at 24 h APF were visualized with an anti-Dlp antibody (red). At this developmental stage, some debris remained but axons were barely visible in controls **(A)**. The glial expression of Orion-CD2 driven by *repo-GAL4* (along with a neutral *UAS-HO1*) induces clear MB pruning defects with many unpruned axons and a thicker dorsal lobe **(B)**. This pruning phenotype is rescued by the overexpression of Drpr in glia **(C)**, indicating an interaction between Drpr and Orion-CD2. *orion* ^*ΔC*^ **(D)** and *drpr* ^*indel3*^ **(E)** pupae both show a strong pruning phenotype, and overexpression of Drpr alone in glia does not induce a pruning phenotype **(F)**. **(G–I)** Models of Drpr and Orion-CD2 interactions in pupae leading to the phenotypes described above. In controls **(G)**, MB Orion and astrocytic Drpr interact, with normal pruning. When a membrane Orion-CD2 is expressed in astrocytes, its titration of Drpr prevents endogenous Orion binding, with visible pruning defects **(H)**. When Drpr is overexpressed in glia along with Orion-CD2, there is a sufficient level of Drpr to interact with both Orion-CD2 and endogenous Orion, and the pruning defects are rescued **(I)**. **(J,K)** Quantitation of the dorsal γ-lobe area at 24 h APF for each condition. *n* values are indicated in the parenthesis and represent the number of MBs visualized. Error bars represent mean ± S.E.M. ****P < 0.0001 (Mann–Whitney U-test). ns, non-significant. Experiments were replicated three times. Scale bars represent 20 μm. Images are z-stacked confocal images. Genotypes are listed in the complementary list of fly strains.

## Results and discussion

Our laboratory published that Drpr was not, or at least not the sole, Orion receptor principally based on the different penetrance of the MB-pruning phenotype in *orion* ^*null*^ (either *orion* ^*1*^ and *orion* ^*ΔC*^) and *drpr* ^*Δ5*^ (Boulanger et al., 2021). We showed here that *drpr* ^*Δ5*^ is not a null allele contrarily to *drpr* ^*indel3*^ (Figure 1). The MB-pruning phenotype of *drpr* ^*indel3*^ was very similar, if not identical, to that of *orion* ^*1*^ with 100% penetrance of the phenotype (n = 36 and n > 20 MBs, respectively), although most of the *drpr* ^*Δ5*^ MBs appeared wild-type, with only a minority of MBs showing unclear axonal debris and unpruned axons, as observed with *UAS-mGFP* under the control of *201Y-G*AL4 (Figures 1A–E). We confirm these results by labeling the MBs with an anti-Dlp antibody, which, in adult, particularly labels the MB γ-neurons (Figures 1F–J). Drpr belongs to the Nimrod family of the phagocytic receptor together with NimC1 and Eater (Melcarne et al., 2019). *NimC1* and *eater* double null mutant flies are deficient in the phagocytosis of all types of bacteria, suggesting that they are the two main phagocytic receptors for bacteria in *Drosophila* (Melcarne et al., 2019). Nevertheless, *NimC1* and *eater* double null mutant flies had no effect on MB remodeling (Figure 1K). This may indicate that phagocytosis of neuronal debris after remodeling requires Drpr as a specialized signaling pathway but does not require the main receptors involved in microbe engulfment. Finally, we showed, through Western blotting, that although *drpr* ^*indel3*^ pupae did not produce any Drpr protein, some Drpr proteins were still present in *drpr* ^*Δ5*^ pupae, confirming the null allele status of *drpr* ^*indel3*^ (Figure 1L). These results are in accordance with *drpr* ^*Δ5*^ being a mutation affecting strongly, but not completely, the transcription of the *drpr* mRNA (Freeman et al., 2003). Therefore, in rare cases when a Drpr protein is produced, in *drpr* ^*Δ5*^ flies, it is a normal-sized protein. Contrarily, *drpr* ^*indel3*^ is a CRISPR mutation affecting the coding sequence, leading to a protein null (Sapar et al., 2018). It is expected that in an exclusive relationship between a ligand and its receptor for a particular process, the null mutant phenotype of each gene has a similar, if not identical, phenotype. This result is a strong indication that the glial phagocytic receptor Drpr functions as a receptor for the neuron-secreted Orion ligand during the MB remodeling process.

Orion is a signal peptide-bearing protein without any transmembrane domain. Therefore, Orion could move in the extracellular space (Boulanger et al., 2021). We wondered if expression of Orion that is permanently tethered to the plasma membrane of otherwise wild-type MB axons could alter Orion function and produce a gain-of-function phenotype. We examined L3 larvae, a stage when the remodeling process has normally not begun, and found that although expression of a wild-type *UAS-orion* transgene in the MBs showed no glial phenotype, expression of *UAS-orion-CD2* dramatically recruited glia inside γ-axon bundles, as monitored by the presence of the Drpr receptor, which serves as a glial marker (Figure 2). We assessed that similar expression levels of Orion-Myc and Orion-CD2-Myc were observed at the tip of the larval dorsal γ-lobes (10604±561 and 11114±842, respectively, n = 25 measures on 5 MBs for each condition, p = 0.84, Mann–Whitney U test). In the *UAS-orion-CD2* transgene, Orion is located on the extracellular side of the CD2 transmembrane domain and could potentially interact with the glial receptor Drpr. Why is there glia infiltration, at the L3 stage, when the *UAS-orion-CD2* transgene, but not the wild-type *UAS-orion* transgene, is used? We can propose two non-exclusive hypotheses. One is that Orion-CD2, contrarily to Orion, is not secreted and therefore is immediately available for the potential protein–protein interaction. Another hypothesis could be a similar model for the MBs and for the C4da neuron dendrite pruning (Ji et al., 2023). The CD2 transmembrane domain contained in Orion-CD2 could mimic an extracellularly exposed molecule through which Orion could bridge Drpr. This molecule could be a glycosaminoglycan (GAG) since the GAG binding is necessary for Orion function (Boulanger et al., 2021) or PS as in the case of the C4da neurons. In L3 larvae, these molecules may not be present, or present but not yet functional. The artificial binding of Orion-CD2 to the γ-axon plasma membrane could circumvent the necessity of Orion binding to GAGs or PS, allowing a premature and likely more stable direct interaction with Drpr.

We then asked whether the gain-of-function phenotype caused by the expression of the *UAS-orion-CD2* transgene in L3 (i.e., abnormal early attraction of glia on the MB) could produce a morphological phenotype later in development. We examined 6 h after puparium formation (APF) and found that contrarily to the wild-type situation, when the *UAS-orion-CD2* transgene was expressed in MBs, the extremities of the lobes were missing. This phenotype is particularly evident in the dorsal lobe and was termed the “headless” phenotype (Figure 3) (Figures 3A–C). We reasoned that if this “headless” phenotype resulted from an interaction between neuronal Orion-CD2 and glial Drpr, it should be rescued in a *drpr* ^*null*^ genetic background. Noteworthy, we observed a complete rescue of the Orion-CD2-induced headless phenotype in *drpr* ^*indel3*^ pupae at 6 h APF (Figure 3D). In addition, we hypothesized that ectopic expression of Drpr in MB axons, where it is not normally expressed, might also rescue the phenotype. Interestingly, we found a clear rescue of the “headless” phenotype under these conditions (Figure 3E). Notably, the lack of Drpr expression (in *drpr* ^*indel3*^ individuals) or the ectopic expression of Drpr alone in MBs did not produce an MB phenotype (Figures 3E,F). These results suggest that ectopic expression of Drpr in the MB neurons titrates Orion-CD2, thus preventing it from an early abnormal interaction with endogenous glial Drpr (Figures 3G–I ). The endogenous glial Drpr receptor is then available to interact normally with endogenous secreted Orion.

We finally asked whether Orion-CD2 could titrate endogenous glial Drpr. To address this, we ectopically expressed Orion-CD2 in glia and examined MB pruning at 24 h APF using an anti-Dlp antibody. Indeed, expression of Orion-CD2 in glia blocked the pruning process compared to the wild-type situation (Figures 4A,B). In addition, the pruning phenotype caused by ectopic expression of Orion-CD2 in glia was rescued when Drpr was simultaneously overexpressed in glia (Figure 4C). These phenotypes suggest that ectopic expression of Orion-CD2 in glia titrates endogenous glial Drpr, thereby preventing it from interacting with endogenous neuron-secreted Orion (Figures 4G,H). In addition, when Drpr is overexpressed in glia together with Orion-CD2, it titrates Orion-CD2, allowing the normal interaction between endogenous glial Drpr and endogenous neuron-secreted Orion (Figures 4D-F,I) and leading consequently to normal pruning.

The requirement of the presence of the Drpr protein to allow the Orion-CD2-induced headless phenotype and the titrating results of Drpr on Orion-CD2 and conversely of Orion-CD2 on Drpr associated to the similar, if not identical, mutant phenotype of both null alleles of *drpr* and *orion* strongly support the conclusion that the neuron-secreted chemokine-like Orion ligand interacts with the glial phagocytic receptor Drpr during MB remodeling in *Drosophila*.

## Data Availability

The raw data supporting the conclusions of this article will be made available by the authors, without undue reservation.
